# Case Reports of Human Monkeypox Virus Infections, Uganda, 2024

**DOI:** 10.3201/eid3101.241269

**Published:** 2025-01

**Authors:** Nicholas Bbosa, Stella E. Nabirye, Hamidah S. Namagembe, Ronald Kiiza, Alfred Ssekagiri, Mary Munyagwa, Arafat Bwambale, Stephen Bagonza, Henry Kyobe Bosa, Robert Downing, Julius Lutwama, Pontiano Kaleebu, Deogratius Ssemwanga

**Affiliations:** Uganda Virus Research Institute, Entebbe, Uganda (N. Bbosa, S.E. Nabirye, A. Ssekagiri, R. Downing, J. Lutwama, P. Kaleebu, D. Ssemwanga); Medical Research Council/Uganda Virus Research Institute and London School of Hygiene and Tropical Medicine Uganda Research Unit, Entebbe (N. Bbosa, H.S. Namagembe, R. Kiiza, P. Kaleebu, D. Ssemwanga); Bwera General Hospital, Bwera, Uganda (M. Munyagwa); Kasese District Local Government, Kasese, Uganda (A. Bwambale, S. Bagonza); Ministry of Health of Uganda, Kampala, Uganda (H.K. Bosa); Uganda Peoples’ Defence Forces, Kampala (H.K. Bosa); Makerere University Lung Institute, Kampala (H.K. Bosa)

**Keywords:** mpox, viruses, sexually transmitted infections, monkeypox virus, human, Kasese District, PCR, next-generation sequencing, Uganda

## Abstract

Mpox is a zoonotic disease caused by the monkeypox virus. We report on human mpox cases in Uganda identified by PCR and confirmed by deep sequencing. Phylogenetic analysis revealed clustering with other clade Ib sequences associated with recent outbreaks in the Democratic Republic of the Congo.

Mpox is a zoonotic disease caused by the monkeypox virus (MPXV), 1 of the 4 orthopoxvirus species that are pathogenic to humans; others include variola virus (the causative agent of smallpox), cowpox virus, and vaccinia virus (*1*). MPXV was initially discovered in monkeys in a Denmark laboratory in 1958 (*2*). Human mpox was identified in 1970 in the Democratic Republic of the Congo (DRC) and is endemic to west and central Africa (*3*). Human-to-human transmission mostly occurs through close contact with infected persons through direct contact with skin lesions, respiratory droplets, contaminated fomites, and sexual contact (*4*). MPXV consists of 2 clades that are subdivided into sublineages: clade I (formerly the Central African or Congo Basin clade) and clade II (formerly the West African clade) (*5*). Clades I and II show ≈0.5% genomic sequence difference (*5*). Clade Ib, a sublineage of clade I, has been associated with recent mpox outbreaks in the DRC and causes more severe disease than clade II (*6*).

The Africa Centres for Disease Control and Prevention and the World Health Organization have declared mpox a Public Health Emergency of Continental Security and of International Concern (*7*,*8*). Since early May 2022, cases of mpox have been reported in countries where the disease is not endemic (*9*). In Africa, Burundi, Cameroon, Central African Republic, Congo, Cote d’Ivoire, DRC, Kenya, Liberia, Nigeria, Rwanda, South Africa, and Uganda have reported new cases in 2024. In light of ongoing MPXV transmission and an increasing number of cases reported in DRC (*9*), we heightened surveillance for MPXV infections at the Uganda Virus Research Institute (UVRI) sentinel surveillance site in Bwera, Kasese District, and at the Mpondwe border point-of-entry in western Uganda. The rationale was to enhance MPXV surveillance through a deliberate and targeted approach to mitigate public health risk for cross-border spillover in areas bordering DRC and Uganda. The study was done as part of the Viral Pathogen Surveillance and Discovery study approved by the UVRI Research Ethics Committee (ref. no. GC/127/908) and the Uganda National Council of Science and Technology (ref. no. HS2543ES).

## The Study

**Figure 1:**
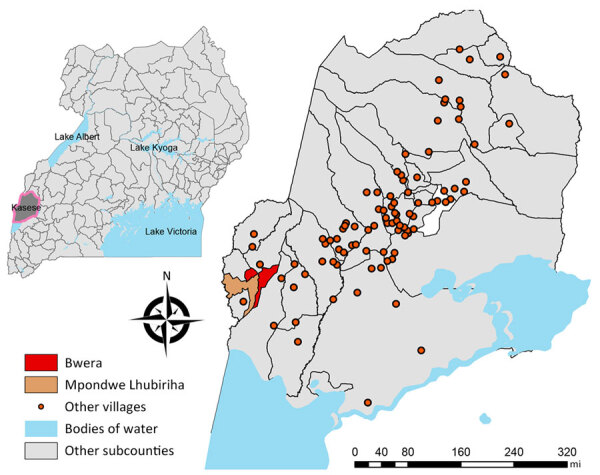
Geography of sampling sites in study of human monkeypox virus infections, Uganda, 2024. Larger map shows sampling sites of Bwera and Mpondwe Lhubiriha in Kasese District. Inset map shows location of Kasese District in western Uganda.

We set up an observatory at Bwera Hospital, a large health facility serving communities in Uganda and the neighboring DRC (Figure 1). Furthermore, we trained health screening teams at the Mpondwe border (Figure 1) in case definition and sample collection. We performed community sensitization to help identify and report suspected cases to strengthen preparedness and response.

During late June–July 2024, we identified 6 suspected mpox cases; patients had signs and symptoms such as skin rash, lymphadenopathy, general malaise, and fever. We collected lesion swab samples in viral transport media and obtained whole-blood specimens from suspected case-patients. Lesion swabs and blood samples were collected from 3 clinically suspected persons (6 specimens); for the other 3 persons, only swabs were collected, for a total of 9 specimens (Table). Samples were transported to UVRI laboratories under cold chain for testing. Skin lesion swab samples are the recommended sample type for laboratory confirmation of MPXV by nucleic acid amplification-based methods. We performed real-time MPXV-specific PCR using Creative-Biogene (https://www.creative-biogene.com) and Roche LightMix Modular (https://www.roche.com) PCR tests on the QuantStudio 7 Real-Time PCR system (Thermo Fisher Scientific, https://www.thermofisher.com).

**Table:** Summary of PCR and genotyping results in study of human MPXV infections, Uganda, 2024*

Case no.	Age/sex	Sample type	Date of sample reception at the laboratory	Date of qPCR	PCR mpox		Date of NGS	UVRI Metagenomics	Dragen Microbial Enrichment Plus
					Roche	Creative Biogene			
1	37 y/F	Blood	2024 Jul 20	2024 Jul 21	ND	ND		ND	ND
		Swab	2024 Jul 20	2024 Jul 21	Positive (Ct 18.29)	Positive (Ct 21.18)	2024 Jul 22	MPXV	MPXV
2	22 y/F	Blood	2024 Jul 20	2024 Jul 21	ND	ND	ND	ND	ND
		Swab	2024 Jul 20	2024 Jul 21	Positive (Ct 35.4)	Negative	2024 Jul 22	MPXV	MPXV
3	1 y/F	Blood	2024 Jul 20	2024 Jul 21	ND	ND	ND	ND	ND
		Swab	2024 Jul 20	2024 Jul 21	Negative	Negative	ND	ND	ND
4	11 mo/M	Swab	2024 Jul 20	2024 Jul 21	Negative	Negative	ND	ND	ND
5	35 y/F	Swab	2024 Jul 20	2024 Jul 21	Negative	Negative	ND	ND	ND
6	Unknown/F	Swab	2024 Jul 20	2024 Jul 21	Negative	Negative	ND	ND	ND

*Case definitions: Clinical criteria for a suspected case are new characteristic rash on the skin, ano-genital or elsewhere on the body, which may include single or multiple lesions or meets one of the epidemiologic criteria and has a high clinical suspicion for mpox. Criteria for probably case: no suspicion of other recent *Orthopoxvirus* exposure and demonstration of the presence of *Orthopoxvirus* DNA by PCR of a clinical specimen. Laboratory criteria: detection of MPXV-specific DNA sequences by PCR and /or sequencing of a clinical specimen. Epidemiologic criteria: >1 of the following epidemiologic links in the last 21 d before symptom onset: reports having contact with a person or persons with a similar appearing rash or who received a diagnosis of confirmed or probable mpox or traveled outside Uganda to a country with confirmed cases of mpox or where MPXV is endemic like the Democratic Republic of Congo or had close or intimate in-person contact with persons in a social network currently experiencing MPXV activity. Ct, cycle threshold; MPXV, monkeypox virus; NGS, next-generation sequencing; ND, not done; qPCR, quantitative PCR.

Samples collected from 2 of 6 clinically suspected patients tested positive for MPXV by PCR; in 1 patient, 2 swab specimens tested positive on both tests, and in the other patient, a swab specimen tested positive on 1 test (Table). One MPXV-positive patient was a 37-year-old female market vendor and saloon owner who was married to a man from DRC and resided in Mpondwe Lhubiriha, Kasese District. She traveled frequently to DRC. The patient had swollen lymph nodes and a skin rash that included nonpruritic generalized papular-vesicular skin eruptions; the rash initially involved the hands but rapidly spread to the rest of the body by day 2 of onset. Onset of symptoms was July 8, 2024, and she visited Bwera Hospital on July 15. The other positive case was in a 22-year-old pregnant woman from DRC who resided in Kamukumbi Village and sought antenatal care at Bwera Hospital. She worked as a hairdresser in Bwera. Symptoms began on July 11 with the sudden onset of pruritic small vesicular eruptions on her skin, which initially involved her hands but spread rapidly to the rest of the body by day 2 of onset. Symptoms resolved within 4 days of onset, and her baby was delivered by caesarean on July 18. She had been exposed to persons with skin rash in her work and to sick poultry but had no recent travel history to the DRC. She also experienced mild fever and lymphadenopathy and had sought care at Bwera Hospital on July 14, where a sample was collected and tested for MPXV. The patient tested positive for MPXV on the Roche PCR but negative on the Creative-Biogene PCR (Table). Both real-time PCRs are developed to detect MPXV clade 1b strains. The other 4 patients tested negative for MPXV on both assays. For positive samples, we performed target enrichment next-generation sequencing using the Viral Surveillance Panel on the MiSeq platform (Illumina, https://www.illumina.com). We analyzed deep sequence reads using UVRI in-house metagenomics analysis (https://github.com/UVRI-BCB/Metagenomics) and DRAGEN Microbial Enrichment Plus (Illumina). High-quality MPXV genomic reads were generated in both samples with >95% genome coverage (99.4% for case 1 and 96.7% for case 2 relative to GenBank accession no. NC_003310.1) (Table).

**Figure 2:**
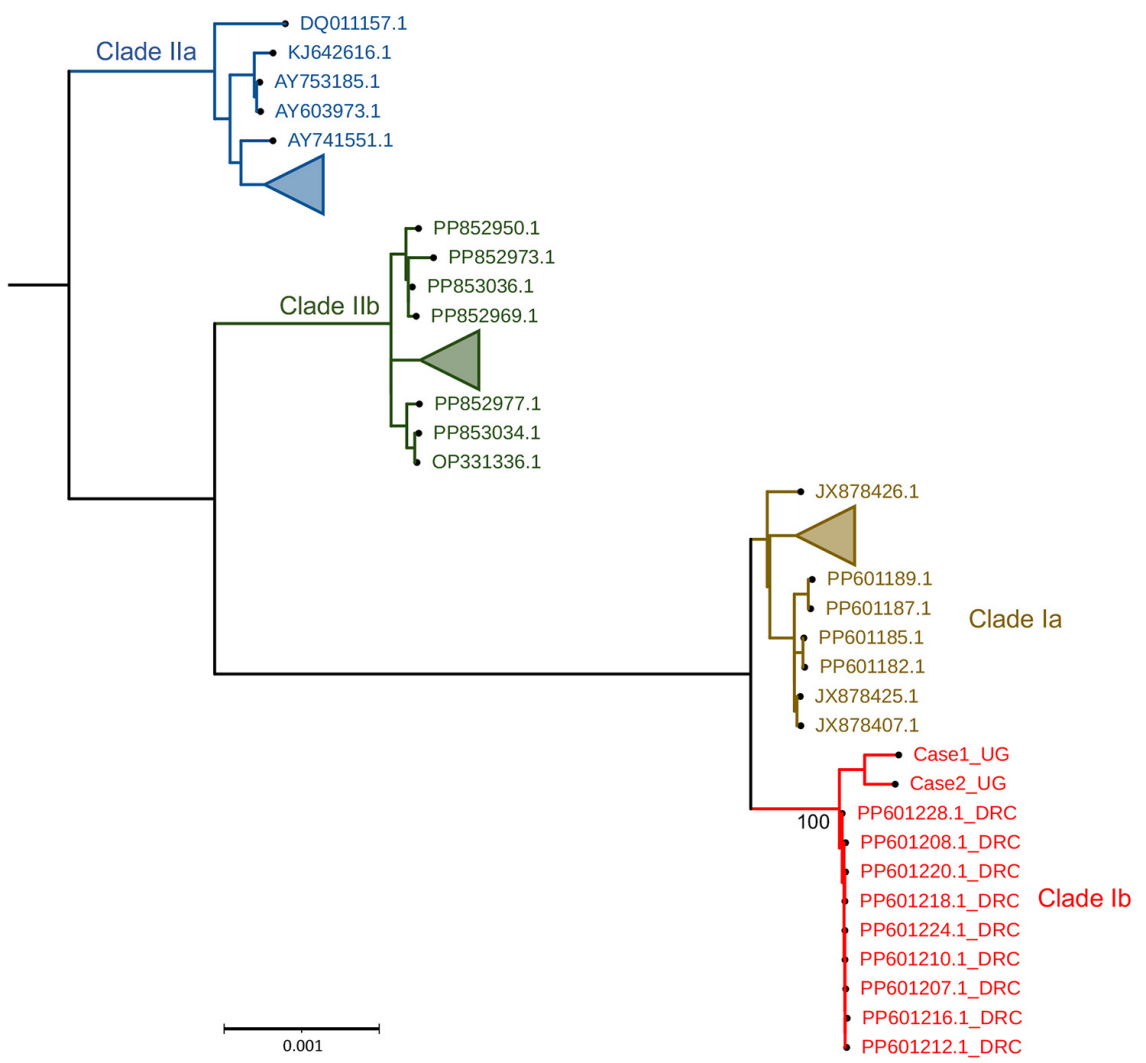
Phylogenetic tree showing clade analysis in study of human monkeypox virus infection, Uganda, 2024. Maximum-likelihood tree was generated using IQ-TREE (https://www.iqtree.org) with 1,000 bootstrap resampling. The monkeypox virus sequences from Uganda (Case1_UG and Case2_UG) clustered with other clade Ib viruses from the Democratic Republic of the Congo (red text). Scale bar indicates number of nucleotide substitutions per site.

We further characterized the viruses as belonging to clade Ib (Figure 2), associated with recent MPXV outbreaks in DRC. Phylogenetic analysis demonstrated that MPXV sequences sampled from Bwera were closely genetically related to other clade Ib sequences from DRC. Findings suggest that the MPXV sequences detected in this report are similar to those associated with the South Kivu outbreak (*6*). Mutation analysis showed that the most mutated genes were OPG164, OPG210, OPG015, and OPG015_dup but also included a D14L (OPG032) gene deletion (Appendix). We deposited the 2 MPXV sequences from Uganda were deposited in the GISAID public database (https://www.gisaid.org; under EpiPox; accession nos. EPI_ISL_19305614 and EPI_ISL_19305615). 

PCR is currently the laboratory standard for diagnosing MPXV infection (*10*). Although no viral load results were available for the 2 cases in Uganda, the cycle threshold value for case-patient 2 (who tested positive by 1 PCR) was 35.4. The number of MPXV sequence reads generated by NGS for case-patient 2 (≈6,000) was comparatively lower than those for case-patient 1 (≈12,000), who tested positive on both assays. This finding could have resulted from low-level viremia in the case-patient 2 sample, which correlated with a higher cycle threshold value, depicting a lower concentration of viral genetic material (*11*). In addition to viral loads, differences in detection could be related to the primer target regions of the virus; the Roche assay targets conserved regions but target regions for the Creative Biogene assay are not disclosed.

## Conclusions

Laboratory testing by PCR and genomic sequencing confirmed the presence of MPXV in 2 patient samples collected from Bwera, western Uganda, associated with outbreaks in DRC. Mpox is no longer a rare disease limited to only endemic countries. Kenya reported its index case in July 2024 from a long-distance trucker who traveled from Uganda (S.K. Langat et al., unpub. data, https://www.biorxiv.org/content/10.1101/2024.08.20.608891v1). In Uganda, surveillance and response is ongoing in Bwera and at sites across the country to identify transmission chains and implement control and prevention measures. Efforts are underway to conduct serosurveys to estimate MPXV prevalence and acquire vaccines. Genomic surveillance is critical to monitor MPXV variants to foster improvements in diagnostics, vaccines, and patient management. 

Appendix Additional information about human monkeypox virus infections, Uganda, 2024
