# Supplementary material for: Case Reports of Human Monkeypox Virus Infections, Uganda, 2024
Source: Emerg Infect Dis. 2025 Jan;31(1):144–8. doi: 10.3201/eid3101.241269 (PMC11682813; doi:10.3201/eid3101.241269)
Supplement: Appendix — Additional information about human monkeypox virus infections, Uganda, 2024 [file 24-1269-Techapp-s1.pdf]

Article DOI: https://doi.org/10.3201/eid3101.241269

EID cannot ensure accessibility for supplementary materials supplied by authors. Readers who have difficulty accessing supplementary content should contact the authors for assistance.

Case Reports of Human Monkeypox Virus Infection, Uganda, 2024

Appendix

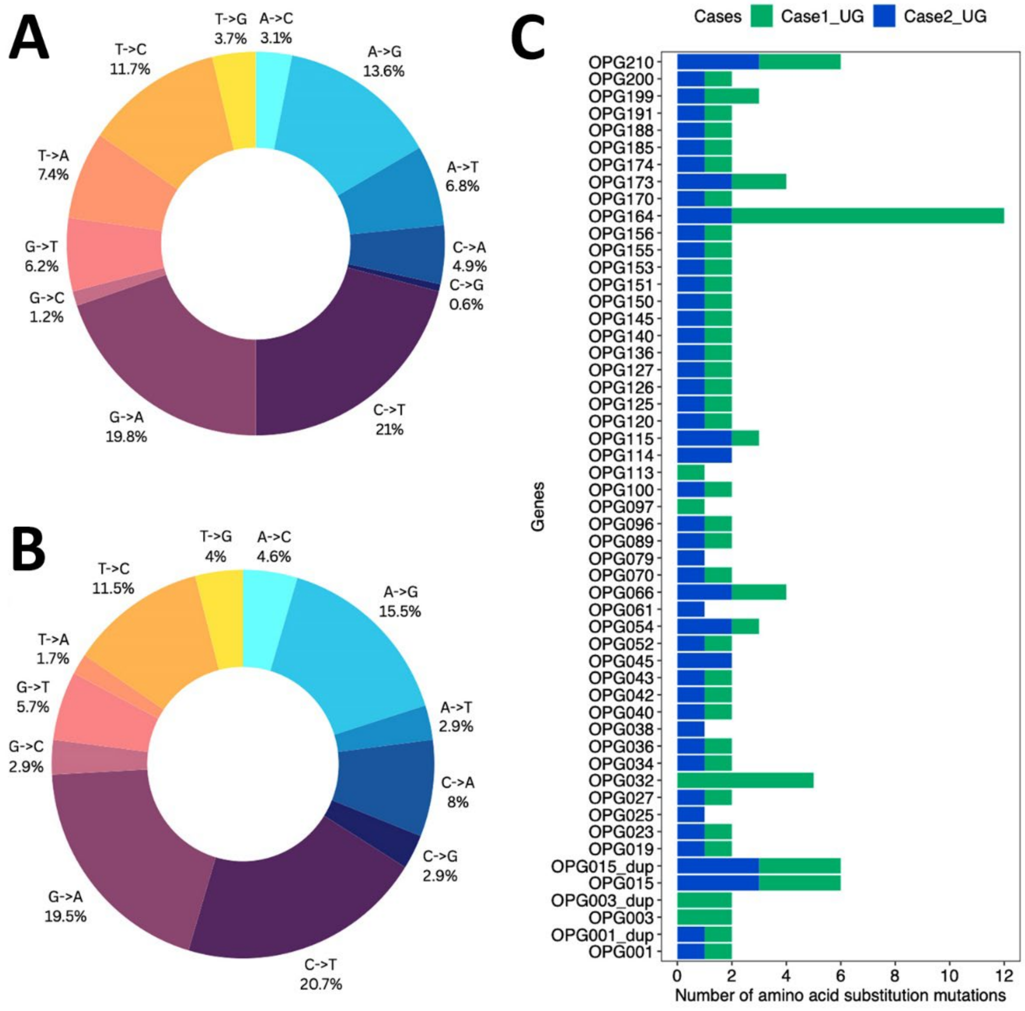

Appendix Figure. Pie charts showing nucleotide mutations for Case2_UG (a) and Case1_UG (b). There was a high abundance of G ->A and C ->T mutations in both sequences. (c) Bar plot showing the number of aa substitution mutations among genes for Case1_UG and Case2_UG.
